# DEADS: Depth and Energy Aware Dominating Set Based Algorithm for Cooperative Routing along with Sink Mobility in Underwater WSNs

**DOI:** 10.3390/s150614458

**Published:** 2015-06-18

**Authors:** Amara Umar, Nadeem Javaid, Ashfaq Ahmad, Zahoor Ali Khan, Umar Qasim, Nabil Alrajeh, Amir Hayat

**Affiliations:** 1 COMSATS Institute of Information Technology, Park Road, Islamabad 44000, Pakistan; E-Mails: amara_t7@yahoo.com (A.U.); ashfaqcomsats@gmail.com (A.A.); amir.hayat@comsats.edu.pk (A.H.); 2 Internetworking Program, FE, Dalhousie University, Halifax, NS B3J 4R2, Canada; E-Mail: zahoor.khan@dal.ca; 3 University of Alberta, AB T6G 2J8, Canada; E-Mail: umar.qasim@ualberta.ca; 4 College of Applied Medical Sciences, Department of Biomedical Technology, King Saud University, Riyadh 11633, Saudi Arabia; E-Mail: nabil@ksu.edu.sa

**Keywords:** depth threshold, dominating set, cooperative routing, sink mobility, underwater wireless sensor networks

## Abstract

Performance enhancement of Underwater Wireless Sensor Networks (UWSNs) in terms of throughput maximization, energy conservation and Bit Error Rate (BER) minimization is a potential research area. However, limited available bandwidth, high propagation delay, highly dynamic network topology, and high error probability leads to performance degradation in these networks. In this regard, many cooperative communication protocols have been developed that either investigate the physical layer or the Medium Access Control (MAC) layer, however, the network layer is still unexplored. More specifically, cooperative routing has not yet been jointly considered with sink mobility. Therefore, this paper aims to enhance the network reliability and efficiency via dominating set based cooperative routing and sink mobility. The proposed work is validated via simulations which show relatively improved performance of our proposed work in terms the selected performance metrics.

## 1. Introduction

Oceans cover about two-thirds of the surface of the earth. They have gained importance because of the hidden resources, military surveillance purposes and underwater environment monitoring. UWSNs have gained attention for these explorations. Typical framework of UWSN is composed of underwater sensor nodes, sink nodes, surface stations, *etc*. Acoustic signals are used for communication in UWSNs due to the relatively low absorption rate in underwater environment. However, continuously varying water temperature, salinity, pressure, density and noise make the underwater environment harsh for signal propagation. Furthermore, the unique characteristics of acoustic signals impose numerous challenges on devising efficient data routing protocols in UWSNs. Sensor nodes in UWSNs consume more energy due to severe environmental conditions where replacement or recharging of battery is not feasible. Bandwidth available for communication is limited (<100 kHz) because of high attenuation and absorption of acoustic signals. Link characteristics like transmission loss, propagation delay and Signal-to-Noise Ratio (SNR) are continuously varying with time and depth. Moreover, BER is also high due to multi-path fading and refraction phenomena. Therefore, improved network lifetime, energy efficiency and high throughput along with reliability of received data stand out as a critical consideration [1] .

Graph theory serves to model different network topologies. It is widely used to devise algorithms for efficient data routing in Wireless Sensor Networks (WSNs). UWSNs involving depth-based routing can be represented by a directed graph (or digraph), *G* = (*V, A*). A digraph is a graph, or set of nodes (*V*) connected by directed edges or arcs (*A*), where the arcs have a direction associated with them. We are considering a digraph for data routing in underwater environment because in depth-based routing data needs to be routed upwards, that is, towards low depth (surface station present on water surface).

Cooperative communication exploits the broadcast nature of wireless transmission and relies on cooperation of nodes in relaying each others' information. It involves the use of a source, relay and destination node. When source node broadcasts any signal it is received by destination node and also overheard by relay node in its sensing range. Relay node then forwards this overheard signal to destination node as a replica of the original signal [1]. Two types of relaying schemes usually implemented at relay nodes are: Amplify-and-Forward (AF) and Decode-and-Forward (DF) [2]. Relay node simply amplifies the signal and transmits it in the former scheme, whereas, received signal is decoded, corrected, recoded and then transmitted in later. Different number of relay nodes can be selected as per application requirement. Cooperative communication is incorporated in UWSNs to improve the network performance in terms of packet delivery ratio and data reliability.

General architecture [3–6] of UWSNs involves static sinks which are usually deployed on water surface. However, static sinks result in hotspot problem in which sensor nodes closer to the sinks tend to die early because of forwarding more packets (packets from nodes that lie far away from sinks), leaving certain area of network completely unmonitored. Hence, it is beneficial to use Mobile Sinks (MSs) to transfer sensed information [7].

In this paper, we present a mathematical model for cooperative routing along with sink mobility in UWSNs. This work is an extension of the work in [8] in which cooperative routing along with sink mobility is investigated at network layer. The proposed mathematical model involves an algorithm to compute the Dominating Set (*DS*) of a given network modeled as a digraph. *DS* formation helps in organizing nodes in a way that is optimal for cooperative routing. *DS* is a subset of vertices *V* such that each vertex in *V* – *DS* is adjacent to at least one vertex in *DS*. Vertices which belong to *DS* are able to perform tasks related to data routing and can serve vertices that are not in the set. *DS* formation reduces communication overhead, increases bandwidth efficiency and decreases energy consumption which ultimately increases network lifetime in UWSNs. Vertices in *DS* efficiently forward data towards MSs which are following pre-defined optimal trajectories.

The rest of the paper is organized as follows. In Section 2, related work along with motivation is presented. In Sections 3 and 4, the channel model and the mathematical model are discussed. Section 5 presents a detailed explanation of our proposed scheme. Section 6 deals with the discussion of simulation results and Section 7 concludes the paper.

## 2. Related Work and Motivation

Cooperative communication takes advantage of the broadcast nature of wireless transmissions in which transmitted signal can be overheard by many unintended sensor nodes. It has been proposed as an alternate mechanism over multi-hop communication for minimizing the effect of fading and other link impairments [1]. Some recent work involving cooperative communication is precisely reviewed here.

An innovative physical layer solution involving cooperative communication is given in [1], where outage probability and capacity expressions are derived for cooperative multicarrier Underwater Acoustic Communication (UAC) systems with AF and DF relaying. Effect of several channel parameters is demonstrated via the derived expressions. Furthermore, they propose a receiver design to mitigate the degrading Doppler effects. In [2], Cooperative UnderWater Acoustic Multiple Input Single Output (CUWA-MISO) using DF is proposed. Virtual antennas are used in this work and their effects on system performance are studied. Each node present in the network uses nearest adjacent node as a virtual antenna in a cooperative manner. This process improves the system performance with the help of spatial diversity. Luo *et al.* [9] explore cooperation at MAC layer and propose a distributed MAC protocol; Coordinated Transmission MAC (CT-MAC) for underwater Multiple Input Multiple Output (MIMO) based network uplink communication. Flooding is avoided by devising a coordination scheme among immediate neighbors for channel competition. CT-MAC also addresses long propagation delay and collision among control packets in UAC. Authors in [10] improved channel efficiency by applying asynchronous cooperative transmission for three dimensional UWSNs. Two typical forwarding schemes: AF and DF are implemented, analyzed and compared. Moreover, an adaptive and hybrid forwarding scheme is also proposed in which each node chooses appropriate forwarding scheme to achieve better BER performance. In COoperative Best Relay Assessment (COBRA) [11], a relay selection criterion for underwater cooperative acoustic networks is developed which requires channel statistical information instead of the instantaneous channel state. COBRA minimizes one-way packet transmission time. Best relay selection algorithm based on COBRA criterion is also proposed in this work. In [12], authors consider cooperative scheme focusing on physical, MAC and network layer. This scheme results in an efficient network operation and reduces the transceiver's complexity. Distance cost and local measurement of channel conditions are considered while selecting destination and potential relay nodes. This scheme enhances reliability by providing diversity gains through intermediate relay nodes.

Network lifetime may be improved by using sink mobility. Some of the protocols that involve sink mobility are briefly stated here.

An adaptive strategy for highly dynamic underwater environment is proposed in [7] that guarantees minimum energy consumption by enforcing sink redeployment. Sink redeployment decision is based on routing information. When there is high current mobility in the underwater environment, nodes use comparatively more power for communication. To overcome this high power usage, sink redeployment procedure starts to find an optimal new location for surface sink. A coordination scheme using sink mobility for data gathering in UWSNs is proposed by authors in [13] that achieve energy efficiency and communication reliability. The shortest wake up time and optimal period of sleep time for sensor nodes are investigated in this work. Furthermore, transmission power control by using received signal strength is introduced in order to decrease energy consumption. In [14], an underwater application model for collecting data with multiple MSs is presented. Three algorithms are presented to accomplish the entire network model. These algorithms achieve reduced energy consumption and lower end-to-end delay. In [15], sink mobility is used to improve network lifetime and energy efficiency in three dimensional UWSNs. An optimal mobile sink strategy is proposed to maximize network lifetime by converting each mobility pattern into a combination of circular motions via mapping. MobiRoute defined by authors in [16] supports sink mobility. Sink's controlled and predictable mobility and pause time are favorable features of MobiRoute. Autonomous Underwater Vehicle (AUV) aided Underwater Routing Protocol (AURP) in [17] minimizes total number of data transmissions by using multiple MSs or AUVs as relay nodes, which results in high packet delivery ratio and low energy consumption. In AUV aided Energy Efficient Routing Protocol (AEERP) for UWSNs [18], MS collects data from gateway nodes. Among all the nodes, gateway nodes communicate with MS. Gateway nodes are selected on the basis of their residual energy.

Localization-free, non-cooperative and cooperative, depth-based routing protocols for UWSNs are stated here.

In [3], a novel routing protocol known as Depth-Based Routing (DBR) is proposed that uses depth of sensor nodes as a routing metric and forwards data towards sink by applying greedy algorithm. In [4], an energy efficient routing protocol, named as Energy Efficient Depth Based Routing (EEDBR) is presented. EEDBR utilizes depth as well as residual energy of sensor nodes as routing metrics so that network lifetime can be improved. Authors in Improved Adaptive Mobility of Courier Nodes in Threshold-Optimized DBR (iAMCTD) [5] propose a forwarding function based routing scheme. It is a localization-free and flooding-based routing protocol for underwater applications. iAMCTD achieves energy conservation by incorporating threshold optimized data routing. CoDBR [6] is cooperative depth based routing protocol in which cooperation is employed at network layer. Cooperative routing increases reliability and throughput of the network. Potential relays are selected on the basis of depth information. Data from source node is cooperatively forwarded to the destination by relay nodes.

In UWSNs, high throughput along with reliability of the received information, reduced delay, low BER and energy conservation are of great concern. Novel routing solutions are therefore required for efficient data routing. Cooperative communication achieves high throughput along with reliable data transfer by reducing BER, however, it results in high propagation delay. Sink mobility reduces propagation delay and achieves energy conservation, however, it does not cater for reliability of received information. In this regard, many cooperative communication protocols [12] are proposed that either investigate the physical layer or the MAC layer, however, the network layer is still unexplored. More specifically, cooperative routing has not yet been jointly considered with sink mobility. Therefore, this work focuses on an efficient mathematical model for cooperative routing along with sink mobility in UWSNs because we are interested in taking advantage of positive aspects of both the techniques along with minimizing their drawbacks.

## 3. Channel Model

Channel model is discussed in this section. Each source node modulates its data using Binary Phase Shift Keying (BPSK) modulation scheme. In water (as a communication medium), the transmitted signal suffers fading and noise. Fading is due to multiple paths traversed by the transmitted signal and noise is due to shipping, turbulence, wind, thermal activities, *etc*. In under water environment, typical considerations involve absence of the dominant component. Thus, we assume Rayleigh fading and subject to noise we assume the AWGN model. Equations (1), (2) and (3) [19] describe relationship between the signals that are transmitted and received by source, relay and destination nodes. *X_s_* is the original signal, *Y_sd_* and *Y_sr_* are signals received at destination and relay nodes via source node. *Y_rd_* is signal received at destination node via relay node. *n_sd_* and *n_sr_* are channel noises existing over source to destination and source to relay links. *n_rd_* represents channel noise existing over relay to destination link. *g_sd_* and *g_sr_* are channel gains existing over source to destination and source to relay links. *g_rd_* represents channel gain existing over relay to destination link. Signals received at relay and destination nodes via source node are represented as:

(1) $$Y_{sr}=X_{s}g_{sr}+n_{sr}$$

(2) $$Y_{sd}=X_{s}g_{sd}+n_{sd}$$

Signal received at destination node via relay node is represented as:

(3) $$Y_{rd}=Y_{sr}g_{rd}+n_{rd}$$

Two independently faded copies (*Y_sd_* and *Y_rd_*) of source's data are combined at destination node by using Maximal Ratio Combining (MRC) as a diversity combining technique [19].

## 4. Mathematical Model

**Figure 1:**
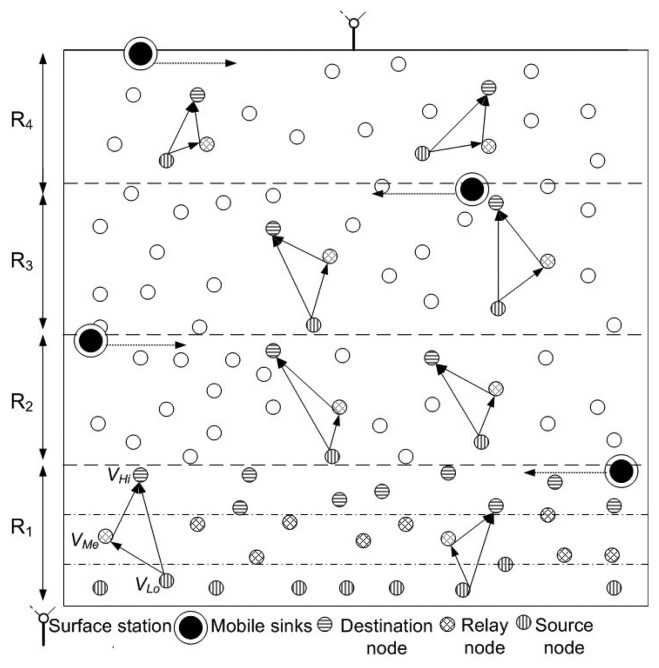
Network model.

In the proposed model, we consider that nodes are randomly deployed in an underwater environment, as shown in Figure 1. All nodes have a predefined transmission range associated with them. Initially, nodes do not possess depth and residual energy information of one-hop neighbors. This information is attained in the broadcasting phase. In this phase, all nodes share depth and residual energy information with one-hop neighbors. Along with this, nodes keep on sharing their residual energy information (at regular time intervals) during the network lifetime. Different rules and concepts that are followed in our algorithm are detailed:

**Rule 1:** Network is divided into 4 distinct regions or sub-graphs (*R_k_* | *k* = 1 − 4) on the basis of depth (*D*), such that, in any subgraph all nodes are one-hop apart and lie within each other's transmission range. Figure 1 signifies rule 1 by showing one-hop apart nodes within each sub-graph.

D(*R*_1_) > *D*(*R*_2_) > D(*R*_3_) > D(*R*_4_)

**Rule 2:** In each *R_k_*, vertices are divided among three distinct sets, *V_Hi_*, *V_Me_* and *V_Lo_* as shown in Figure 1, where

- *V_Hi_* are high depth vertices in a sub-graph
- *V_Me_* are medium depth vertices in a sub-graph
- *V_Lo_* are low depth vertices in a sub-graph

A *DS* is formed for each *R_k_* on the basis of a certain criterion. We define *DS* with respect to this work as;

*DS*: *DS* is a subset of vertices *V* of any *R_k_* such that every vertex not in the *DS* is adjacent to at least one vertex in *DS*.

*DS* for cooperative routing is computed on the bases of depth and residual energy information of sensor nodes. Nodes having lowest depth and highest residual energy in each *R_k_* form a *DS* such that all the other nodes in that *R_k_* are connected to at least one node in *DS*.

**Figure 2:**
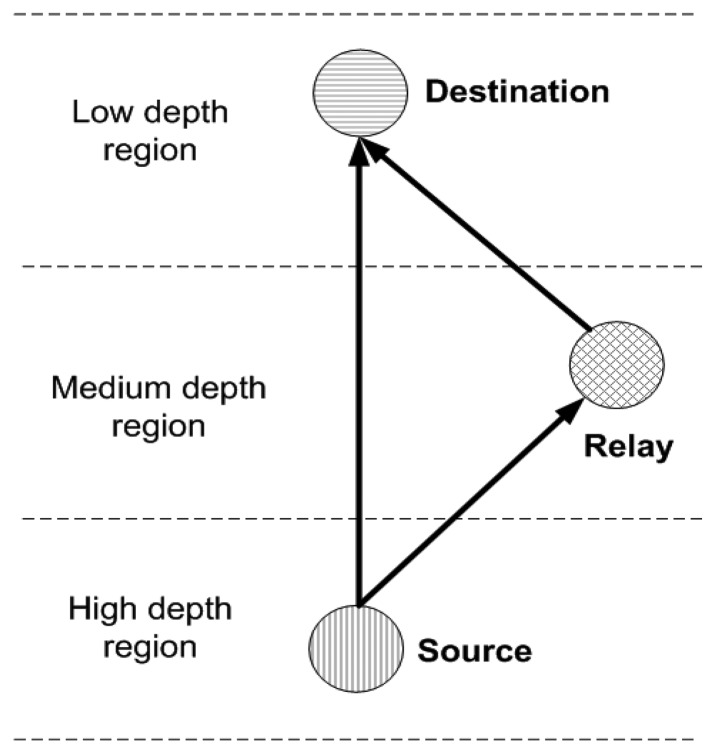
Rule 3: *D_D_* <*D_R_* <*D_S_*.

**Rule 3:** Source, relay and destination nodes in any *R_k_* belong to *V_Lo_*, *V_Me_* and *V_Hi_* such that;

*D_D_* < *D_R_* < *D_S_* where *D_D_, D_R_* and *D_S_* represent depth of destination, relay and source nodes respectively. This relation shows that source, relay and destination nodes are present at highest, medium and lowest depth regions in any *R_k_*, respectively. Rule 3 is shown in Figure 2. We assume that MSs are periodically transmitting beacon signals because of which nodes are aware of their mobility pattern and are forwarding data accordingly.

In wireless transmission, flooding is a routing process in which each incoming data packet is sent via every outgoing link except the one from which it was received. Flooding results in duplication of data and thus wastes resources of the network. In depth-based routing, depth threshold (*d_th_*) is used to avoid flooding by limiting the number of outgoing links for each incoming data packet.

DBR [3], EEDBR [4], iAMCTD [5] and CoDBR [6] are existing depth-based routing protocols in UWSNs. In DBR, EEDBR and CoDBR, *d_th_* is pre-defined and remains fixed throughout the network lifetime. In iAMCTD, *d_th_* is selected and varied according to the network density information which requires exchange of control packets at regular time intervals. This exchange acts as an overhead and wastes network resources. We present a way for varying *d_th_* that minimizes this huge control packet exchange. In our work, nodes just require the residual energy information of one-hop neighbors.

**Rule 4:** Value of *d_th_* is selected and varied according to the number of alive neighbors of a node, *i.e.*,

(4) $$\forall i\in V:d_{th_{i}}(t)\propto N_{i}(t)$$

where, *N_i_*(t) represents alive neighbors of *i_th_* node at time instant *t*

(5) $$N_{i}=N(i)=j|(i,j)\in A$$

**Figure 3:**
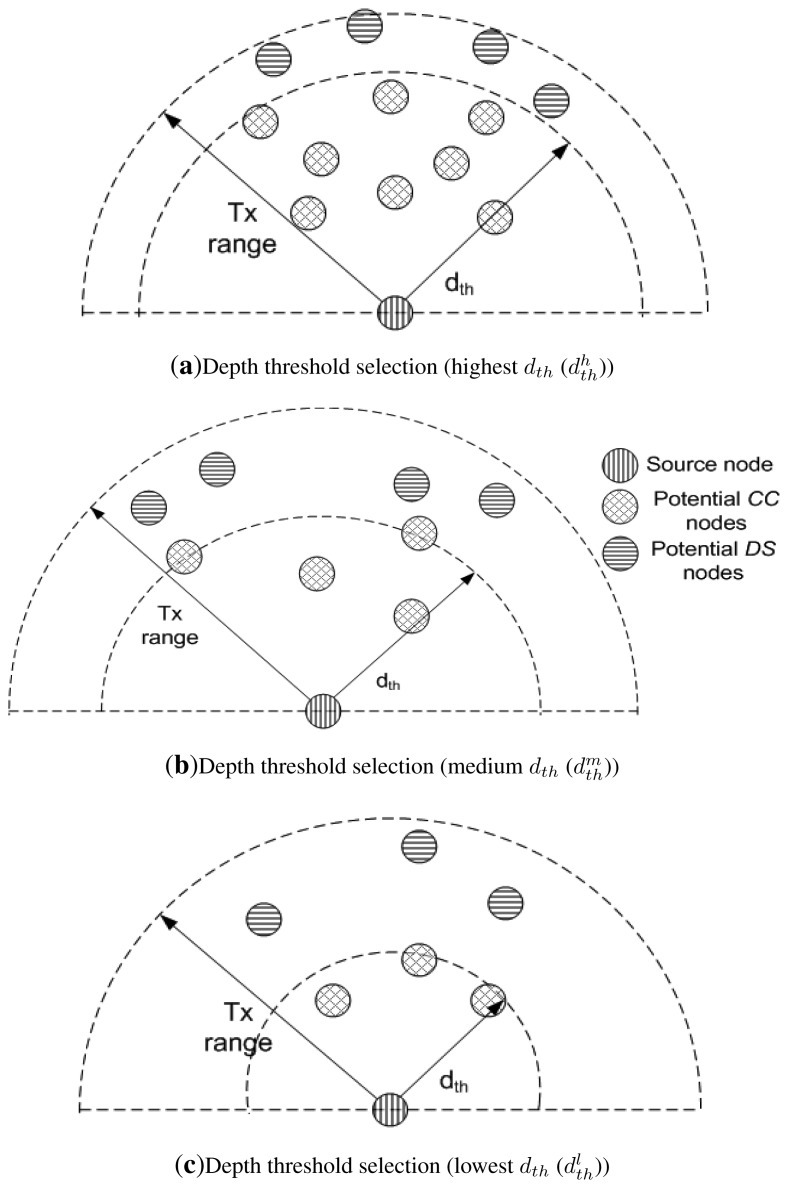
Depth thresholds based on number of neighbors

Increase in *N* leads to increased *d_th_* and decrease in *N* leads to decreased *d_th_*. This relation signifies that the greater the number of alive neighbors, higher is the value of *d_th_* and vice versa. In this way, nodes just require one-hop neighbor information instead of complete network information. *d_th_* plays an important role in selection of destination and relay nodes. Neighbors of a node are distinguished as potential *DS* and Cooperative Connector (*CC*) nodes on the basis of *d_th_* as shown in Figure 3. Destination and relay nodes belong to *DS* and *CC* node sets.

As shown in Figure 3a, number of nodes are present within the transmission range of source node. Due to this high node density its *d_th_* is high ( $d_{th}^{h}$). As node density within the transmission range decreases, *d_th_* also decreases. $d_{th}^{m}$ and $d_{th}^{l}$ show the decreased *d_th_* in Figure 3b,c.

**Rule 5:** Neighbor set of an *i_th_* node is represented as

(6) $$N(i)=N_{{\text{outd}}_{th}}(i)\cup N_{{\text{ind}}_{th}}(i)$$

where

- *N_outd_th__*(*i*) represents neighbors of an *i_th_* node that lie outside the boundary specified by *d_th_* as shown in Figure 3 as potential *DS* nodes.
- *N_ind_th__* (*i*) represents neighbors of an *i_th_* node that lie inside the boundary specified by *d_th_* as shown in Figure 3 as potential *CC* nodes.

(7) $$N_{{\text{outd}}_{th}}\in V_{Lo},N_{{\text{ind}}_{th}}\in V_{Me}$$

Equation (7) signifies that *N_outd_th__*(*i*) and *N_ind_th__*(*i*) are low and medium depth vertices in any *R_k_*, respectively.

**Rule 6:** For each *R_k_*, the potential *DS* nodes are

(8) $$DS_{k}\subset N_{{\text{outd}}_{th}}(V_{Hi})\forall V_{Hi}\in k$$

Initially, for any particular *R_k_*, lowest depth one-hop neighbors (*N_outd_th__*) of vertices represented by *V_Hi_* are considered eligible for inclusion in the *DS*. Smaller the size of *DS*, lesser is the message overhead and network routing efficiency is also improved. In order to reduce the size of *DS*, nodes are further pruned, using their residual energy as a pruning parameter. After pruning in each *R_k_*, lowest depth nodes possessing highest energy are present in *DS*.

**Rule 7:** The lowest depth and highest energy *DS* nodes are the destination nodes for cooperative routing. Whereas, Probability of selection (*Ps*) of any node *i* belonging to *V_Lo_* as *DS* node is

(9) $$\forall i\in V_{Lo}:\{\begin{array}{l} Ps\propto R_{e}(i)\\ Ps\propto 1/D(i) \end{array}$$

Hence, Selection Parameter for *DS* nodes, (*SP_DS_*), is a function of nodes' depth and residual energy:

(10) $$SP_{DS}=f(D,Re)$$

*SP* for *DS* nodes at time *t, SP_DS_*(*t*) is given as:

(11) $$SP_{DS}(t)=R_{e}(t)/D(t)$$

where

**R**_e_(**t**): Residual energy of any *DS* node at time *t* in joules

**D**(**t**): Depth of any *DS* node at time *t* in meters

The higher the value of *SP_DS_*(*t*) for any particular node, the greater the chance of its inclusion in *DS* and vice versa. *DS* node set recalculation is performed at regular time interval, *t* = 120 s, so that the nodes having minimum depth and high energy are selected as *DS* nodes. In this way, chances of data reception at MS are increased and a balanced energy consumption is involved in the network throughout the network operation.

**Rule 8:** The highest energy one hop neighbors (belonging to *N_ind_th__*) of vertices represented by *V_Hi_* are considered eligible to be used as *CCs. CCs* are the relay nodes used in cooperative routing. They connect source nodes with the *DS* nodes via an alternate path.

For each *R_k_*, *CC* nodes are:

(12) $$CC_{k}\subset N_{{\text{ind}}_{th}}(V_{Hi})\forall V_{Hi}\in k$$

**Rule 9:** The highest energy nodes are the *CCs* for cooperative routing. Where, *Ps* of any node *i* belonging to *V_Me_* as *CC* node is:

(13) $$\forall i\in V_{Me}:Ps\propto R_{e}(i)$$

Hence, Selection Parameter for *CC* nodes, (*SP_CC_*), is a function of nodes' residual energy:

(14) $$SP_{CC}=f(R_{e})$$

*SP* for the *CC* at time *t* is

(15) $$SP_{CC}(t)=R_{e}(t)$$

The larger the value of *SP_CC_*(*t*) for any particular node belonging to *V_Me_*, the greater the chances of its inclusion in *CC* node set and vice versa. *CC* node set recalculation is performed at regular time interval, *t =* 120 s, so that high energy nodes are selected as *CC* nodes and a balanced energy consumption is involved in the network throughout the network operation.

**Rule 10:** All *CC* and non-*CC* medium depth vertices in each *R_k_* forward their data towards non-*DS* low depth vertices directly or in a cooperative manner (depending on node availability).

(16) $$\forall i\in V_{Me}|i\in CC_{k},R=j\in V_{Me}|j\notin CC_{k}\&D=l\in V_{Lo}|l\notin DS_{k}$$

(17) $$\forall i\in V_{Me}|i\notin CC_{k},R=j\in V_{Me}|j\notin CC_{k}\&D=l\in V_{Lo}|l\notin DS_{k}$$

Similarly, all non-*DS* low depth vertices in any sub-graph *k* forward their data towards MSs directly or in a cooperative manner (depending on node availability). For any particular *R_k_*;

(18) $$\forall i\in V_{Lo}|i\in DS_{k},D=MS\&R=j\in V_{Lo}|j\notin DS_{k}$$

(19) $$\forall i\in V_{Lo}|i\notin DS_{k},D=MS\&R=j\in V_{Lo}|j\notin DS_{k}$$

where *D* and *R* specify destination and relay nodes. For this specific scenario, *D* and *R* are used instead of *DS* and *CC* due to the reason that *D* and *R* do not belong to *DS* or *CC* node set.

*DS* recalculation is performed at regular time interval, *t* = 120 s, in order to achieve balanced energy consumption throughout the network operation. The nodes require residual energy information in DS re-calculation. However, in contrast to the existing iAMCTD, the information exchange in our proposed work is limited to one-hop neighbors only. In this way, the overhead is reduced.

**Figure 4:**
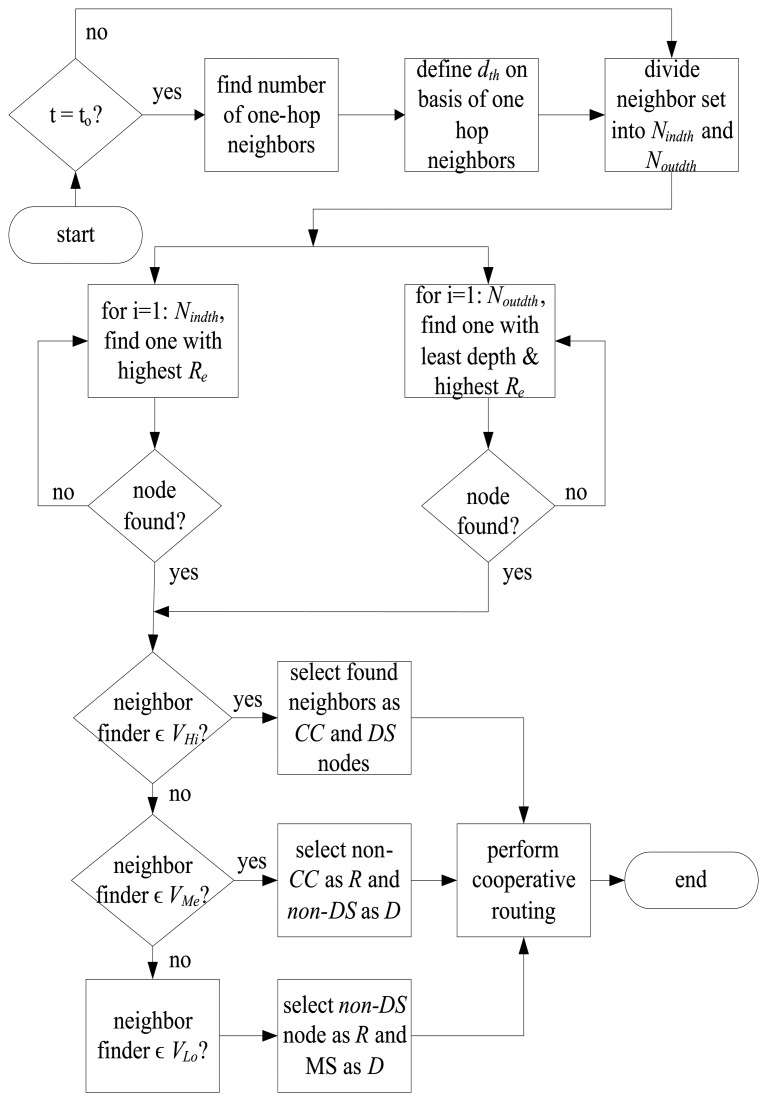
Selection of relay and destination nodes.

The flow chart in Figure 4 shows the selection of relay and destination nodes which is then followed by cooperative routing. All nodes find their one-hop neighbors at regular time intervals, *t_o_* and define their *d_th_* by following rule 4. If a node does not possess any neighboring node it goes in the sleep mode in which it does not perform transmission/reception. After selection of *d_th_*, neighboring nodes are differentiated among *N_ind_th__* and *N_outd_th__* according to rule 5. Nodes belonging to *V_Hi_* use rules 6–9 for the selection of *CC* and *DS* nodes. *CC* node belongs to *N_ind_th__* and possesses the highest *R_e_*, whereas, destination node is one that belongs to *N_outd_th__* and possesses the highest *R_e_* and least depth. Nodes belonging to *V_Me_* and *V_Lo_* follow rule 10 for selection of relay and destination nodes. This is because all the nodes that belong to *V_Me_* and *V_Lo_* might not act as *CC* or *DS* nodes, however, all of them have to forward their data to mobile sink and they forward it by following rule 10. If the neighbor finder node belongs to *V_Me_* of any particular sub-graph, then it selects any non-*CC* node as a relay and non-*DS* node as a destination node. On the other hand, if the neighbor finder node belongs to *V_Lo_* of any particular sub-graph, then it selects any non-*DS* node as a relay, MS as a destination node and forwards data cooperatively.

Now, let us consider a special case of a node which does not find its neighbors. How will it communicate? Well, the chances of the occurrence of this special case are very rare due to two reasons: (i) nodes are densely deployed; and (ii) all the nodes uniformly consume energy. However, near the end of network lifetime, if this situation arises then we handle it via two approaches; (i) if the node is within the communication range of the mobile sink then it directly communicates with it; and (ii) if the node is not within the communication range of the mobile sink, then it remains disconnected from the network and is considered as dead.

## 5. DEADS: The Proposed Scheme

Detailed discussion of the proposed protocol (DEADS) is given in the following subsection. Data sensing and routing example explains the selection of relay and destination nodes in a sub-graph.

### 5.1. Data Sensing and Routing

**Figure 5:**
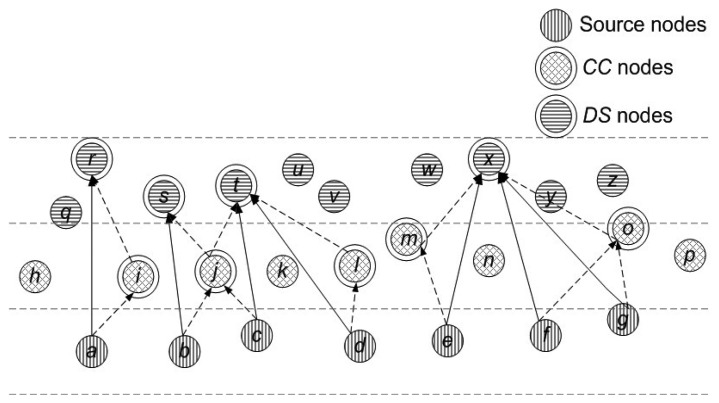
Selection of *DS* and *CC* nodes in a sub-graph.

Let us consider a particular sub-graph as shown in Figure 5 to describe the construction of *DS*. In this example, depth and energy aware *DS* construction for a particular sub-graph is shown. For any vertex *a*, we represent its neighbor set as; *N*(*a*) = *b* ∊ *V* | *a*, *b* ∊ *A* where *b* ∊ *n* (*n* represents total number of nodes in a network).

Initially, each source node finds the number of its one-hop neighbors and selects *d_th_* on its basis. *d_th_* selection process in shown in Algorithm 1. Network lifetime is represented in terms of seconds. *d_th_* recalculation is performed at regular intervals (120 s).

Once a node becomes aware of its *d_th_*, it divides its neighbor set into two distinct sets (*N_outd_th__* and *N_ind_th__*) as shown by the given expressions. In this sub-graph, eligible *DS* nodes are *N_outd_th__* and the eligible *CC* nodes are *N_ind_th__*. For example, node a has nodes *h*, *i*, *j*, *q*, *r* and *s* within its transmission range. After finding its *d_th_* node *a* divides its neighbor set into two sets, finds *CC* and *DS* nodes by following rules 6 to 9 and perform cooperative routing. Node *b*, *c*, *d*, *e*, *f* and *g* follow the same procedure as followed by node *a*. Whereas, all the non-*CC* and non-*DS* nodes forward data by following rule 10.

*N*(*a*) = *h*, *i*, *j*, *q*, *r*, *s*, *N_ind_th__*(*a*) = *h*, *i*, *j*, *N_outd_th__*(*a*) = *q, r, s*,

*N*(*b*) = *i*, *j*, *k*, *r*, *s*, *t*, *N_ind_th__*(*b*) = *r*, *e*, *h*, *i*, *N_outd_th__*(*b*) = *r*, *s*, *t*,

*N*(*c*) = *j*, *k*, *i*, *l*, *s*, *t*, *u*, *v*, *N_ind_th__*(*c*) = *j*, *k*, *i*, *l*, *N_outd_th__*(*c*) = *s*, *t*, *u*, *v*,

*N*(*d*) = *k*, *l*, *m*, *t*, *u*, *v*, *N_ind_th__*(*d*) = *k*, *l*, *m*, *N_outd_th__*(*d*) = *t*, *u*, *v*,

*N*(*e*) = *l*, *m*, *n*, *w*, *x*, *y*, *N_ind_th__*(*e*) = *l*, *m*, *n*, *N_outd_th__*(*e*) = *w*, *x*, *y*,

*N*(*f*) = *m*, *n*, *o*, *w*, *x*, *y*, *z*, *N_ind_th__*(*f*) = *m*, *n*, *o*, *N_outd_th__*(*f*) = *w*, *x*, *y*, *z*,

*N*(*g*) = *n*, *p*, *o*, *x*, *y*, *z*, *N_ind_th__*(*g*) = *p*, *n*, *o*, *N_outd_th__*(*g*) = *x*, *y*, *z*,


**Algorithm 1** Depth Threshold Selection
*t_o_* ← predefined time interval in seconds*T_max_* ← Network lifetime in seconds*N* ← Number of a node's neighbors (*N*_1_ > *N*_2_)*D_th_* ← Depth threshold*D_th_*_1_, *D_th_*_2_, *D_th_*_3_ ← Optimal values of depth threshold (*D_th_*_1_ > *D_th_*_2_ > *D_th_*_3_)**for**
*T* = 1 : *t_o_* : *T_max_*
**do** **if**
*N* ≥ *N*_1_
**then**  *D_th_* = *D_th_*_1_ **else if**
*N*_1_ > *N* ≥ *N*_2_
**then**  *D_th_* =*D_th_*_2_ **else if**
*N* < *N*_2_
**then**  *D_th_* = *D_th_*_3_ **end if****end for**


Traditional routing schemes [3,4,6] consume surplus energy due to transmission of redundant data and retransmission of critical data. We exploit threshold based data sensing in which data is transmitted only when certain predefined thresholds are met. Threshold values depend on the required application. Algorithm 2 describes data sensing and routing procedure. After nodes become aware of their neighbors, they start sensing the specified environmental attribute and forward the sensed data by using cooperative routing.


**Algorithm 2** Data Sensing and Transmission
*S* ← Sensed attribute*H_th_* ← Hard threshold*S_th_* ← Soft threshold*P* ← Data packet*R_e_* ← Residual energy*F* ← Flag**if**
*S* ≥ *H_th_* AND *R_e_* > 0 **then** send P set F = 1**else if**
*S_th_* ≤ *S* < *H_th_* AND *R_e_* > 0 **then** send P set F = 0**else** no transmission**end if**


According to rule 6 (Equation (8)), *DS* is formed and by utilizing *SP_DS_* given in rule 7 (Equations (9), (10) and (11)) each node selects an appropriate destination node that belongs to the *DS* nodes. Similarly, according to rule 8 (Equation (12)), *CC* set is formed and by utilizing rule 9 (Equations (13), (14) and (15)) each node selects an appropriate relay node that belongs to the *CC* nodes. *CC*, non-*CC*, *DS* and non-*DS* nodes also forward their data by utilizing rule 10 (Equations (16), (17), (18) and (19)). In this way, the most optimal destination and relay nodes are selected cooperative routing as shown in Figure 5. *DS* nodes and non-*DS* destination nodes implement MRC on received copies of the data and forward it to MS in its vicinity.

In order to indicate the severity of sensed attribute, flag is introduced. At the on-shore data center, the range of the sensed value can be interpreted just by viewing the flag field. For example, if the sensed attribute is temperature, then “1” indicates that the temperature is very high and appropriate measures must be readily adopted whereas “0” indicates that temperature is high but within an acceptable limit. We have implemented threshold based routing (reactive routing) to conserve network energy. In this type of routing, data is transmitted if the thresholds are met. On the other hand, if data transmission is based on energy then all the nodes will be transmitting all the time (till they are alive) whether it is critical data or not. In this way, relatively more energy will be consumed as compared to the threshold based data transmissions.

### 5.2. Sink Mobility

Conventional UWSN models assume static sink(s), usually present at the surface of water. The sensor nodes forward their data to the sink(s) via multi-hopping. Due to multi-hop communication, the chances of data being corrupted due to link impairments were high. In order to fix this drawback, we use mobile sink in UWSNs. These mobile sinks (also called mobile nodes) move on pre-defined paths and collect data from sensor nodes which are static. In this way, multi-hop communication is significantly reduced. On the other hand, rules are related to the sensor nodes to select their respective relay and destination nodes for cooperative routing. Mobile sinks move on the predefined path, and stop at sojourn locations at every predefined lap of time and collect data from DS nodes in their vicinity. Thus, the idea is to provide alternate paths via relay nodes for assuring transmission to the sink. The mobility patterns are discussed in detail in the following subsections.

#### 5.2.1. Linear Mobility Pattern

**Figure 6:**
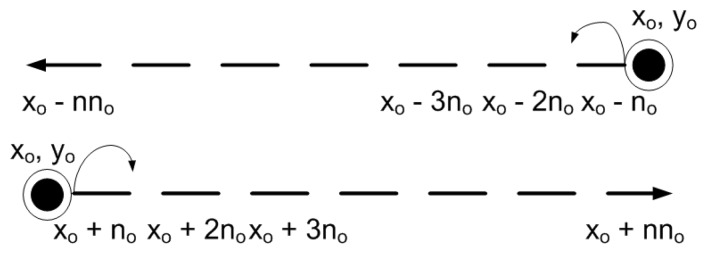
Specifications of linear mobility path followed by MSs.

**Figure 7:**
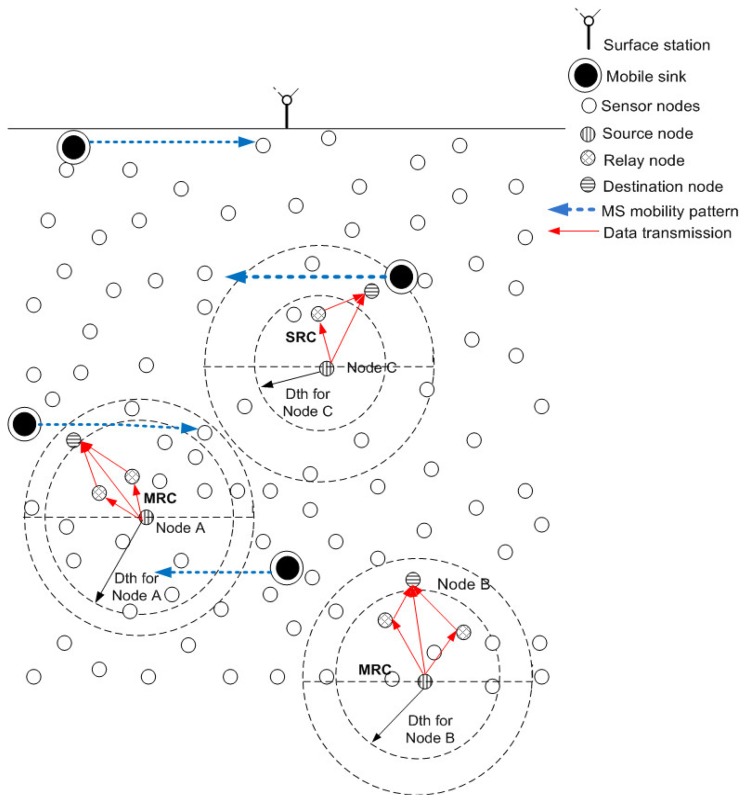
Data routing in the network with linear sink mobility pattern.

MSs follow a horizontally varying linear mobility pattern. MSs are present at different depths (*y_o_*) that remain same throughout the network lifetime. They move either leftwards or rightwards to cover the entire network. MSs change their position after predefined time interval. New position of MSs after a predefined time interval is represented as:

(20) $$x_{MS_{L}}=x_{o}-n_{o}$$

(21) $$x_{MS_{R}}=x_{o}+n_{o}$$

(22) $$y_{MS_{L}}=y_{MS_{R}}=y_{o}$$

where Equations (20), (21) and (22) indicate *x* and *y* dimensions of MSs that are moving leftwards or rightwards. Figure 6 shows the linear mobility path followed by each MS. *n_o_* represents horizontal shift at each predefined time interval and its value can be adjusted according to network requirements. Figure 7 shows data routing in the network involving linear sink mobility pattern. Data is routed from high depth to low depth region according to the rules defined in Section 4. After finding *d_th_*, nodes find appropriate *CC* or *R*, *DS* or *D* nodes and then forward data in a cooperative manner. Nodes forward data by using either Single Relay Communications (DEADS-SRC) or Multiple Relay Communications (DEADS-MRC, two relay nodes in our case). As shown in Figure 7, node *A* and *B* are forwarding data towards the preselected next-hop (*DS* or *D*) node via MRC, whereas, node *C* forwards data via SRC.

#### 5.2.2. Elliptical Mobility Pattern

Each MS follow an elliptical mobility pattern (with vertical major-axis) represented by equation:

(23) $${\left(x/b\right)}^{2}+{\left(y/a\right)}^{2}=1$$

MSs move either Clock-Wise (*CW*) or Counter Clock-Wise (*CCW*) to cover the entire network. MSs change their position after regular time interval. New position of MSs represented by their *x* and *y* dimensions is given by:

(24) $$x_{MS_{CW,\text{CCW}}}=x_{o}+a\times \cos(\theta )$$

(25) $$y_{MS_{CW,\text{CCW}}}=y_{o}+b\times \sin(\theta )$$

**Figure 8:**
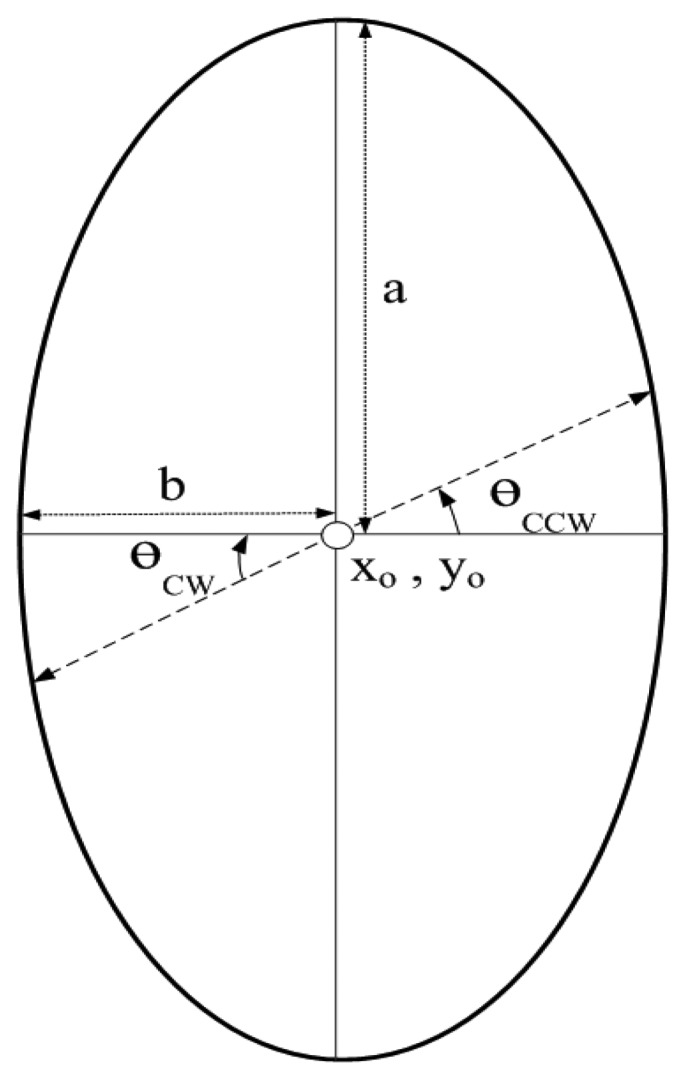
Specifications of elliptical mobility path followed by MSs.

**Figure 9:**
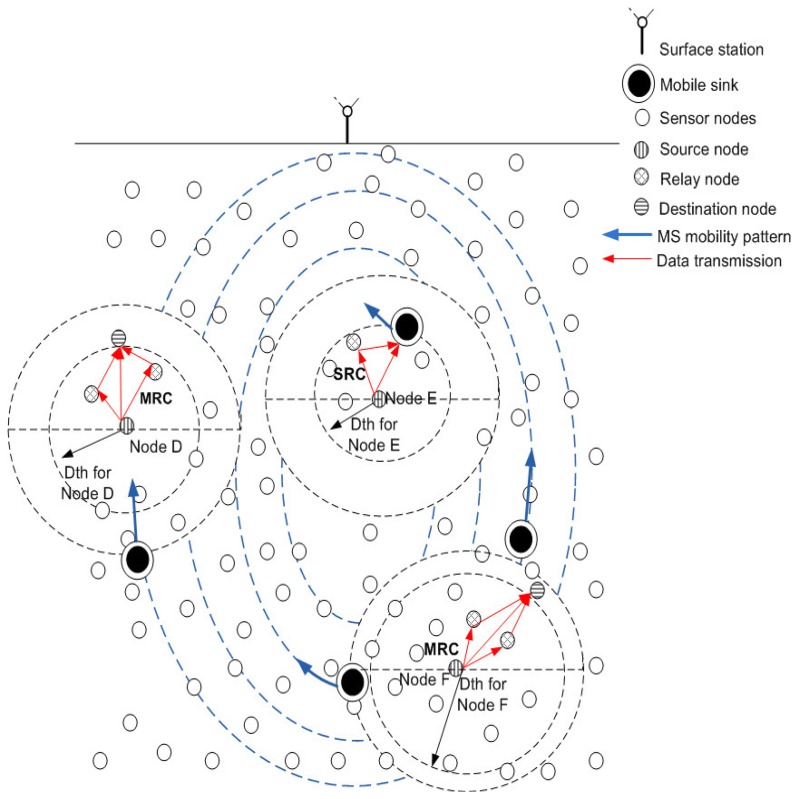
Data routing in the network with elliptical sink mobility pattern.

Figure 8 shows the elliptical mobility path followed by each MS. *x_o_* and *y_o_* represent the center coordinates of each elliptical path which are the same for each ellipse. *a* and *b* represent semi-major and semi-minor axis of each elliptical pattern, where; *a* = 2*b*. Values of *a* and *b* are different for each elliptical path. *θ* represents the angle which is varied for clock-wise or counter clock-wise movement. After each predefined time interval, *θ* is incremented or decremented by a certain value for counter clock-wise or clock-wise movement of MSs. Value of *θ* can be adjusted according to the network requirements. Figure 9 shows data routing in the network in which MSs follow an elliptical mobility pattern. Data is routed from high depth to low depth region according to the rules defined in Section 4. All the nodes find *d_th_*, find appropriate *CC* or *R, DS* or *D* nodes and then forward data by using cooperative manner. Nodes forward data by using either DEADS-SRC or DEADS-MRC. As shown in Figure 9, node *D* and *F* are forwarding data towards the preselected next-hop (*DS* or *D*) node via MRC, whereas, node *E* forwards data directly towards MS via SRC.

## 6. Simulation Results and Discussions

We validate and evaluate our proposed scheme via simulations. We compare DEADS-SRC and DEADS-MRC with existing depth-based routing protocols; DBR, EEDBR, CoDBR and iAMCTD. The selected existing routing protocols have assumed 225 nodes. Thus, for the sake of fair comparison, we have also assumed the same number of nodes. Thus, the 225 sensor nodes are randomly deployed in underwater area of 500 m × 500 m × 500 m. Four MSs are deployed under water at different depths and they are following linear and elliptical mobility patterns. Each sensor node is equipped with an initial energy of 5 Joules and is having a fixed transmission range of 100 m. Size of data packet and control packet is 200 byte and 8 byte respectively. LinkQuest UWM1000 [20] acoustic modem is used having 10 kbps bit rate. Power consumption of a node in transmitting, receiving and in idle modes is 2 W, 0.1 W and 10 mW, respectively. In iAMCTD, there are 4 static sinks and 4 courier nodes for data reception, however, in the proposed protocol only 4 MSs are present. We excluded 4 static sinks present in iAMCTD in order to make it comparable with our proposed protocol. Due to water currents, the sensor nodes exhibit slight horizontal drift but their depth remains unchanged. Moreover, the routing protocol only takes depth of nodes into consideration that is why we assume static nodes. On the other hand, mobile sinks follow predefined elliptical and horizontal trajectories. We also assume the same underlying MAC protocol as in DBR [3], which uses the MAC protocol used in [21]. This, MAC protocol is based on CSMA. Whenever, a node has packets to send, it senses the channel, and then broadcasts the packet(s) if free channel is found. In case of busy channel, it uses a back-off algorithm (with 4 maximum back offs) to contend the channel. The data rate is set to 500 kbps, and the propagation speed of acoustic signals is set 1500 m/s.

DBR, EEDBR and CoDBR are proactive routing (continuous data routing) protocols, whereas, iAMCTD and DEADS are reactive routing (data routing occurs only when certain thresholds are met) protocols. An analysis of the proposed and existing protocols with respect to different parameters is given in Table 1. DBR and CoDBR consider depth, EEDBR considers depth and residual energy and iAMCTD considers link's state in terms of SNR, depth and residual energy of the forwarding nodes as an ultimate forwarding node determining factor. Whereas, in DEADS, depth and residual energy of the forwarding nodes are considered as forwarding node determining factor. To evaluate the performance of DEADS following evaluation metrics are considered:

**Network lifetime:** Network lifetime is the duration up till which all nodes in the network run out of energy. At any time instant, alive nodes represent the number of nodes which have sufficient residual energy for data transmission.

**Residual energy:** It is the total unexpended energy possessed by the network per time instant.

**Throughput:** It is the total number of data packets received at sink per unit time.

**Packet drop:** It shows the number of dropped packets out of total number of packets generated by nodes.

**Packet Acceptance Ratio (PAR):** It is defined as the ratio of total number of packets received at sink to the total number of packets sent to the sink at regular time interval.

**Figure 10:**
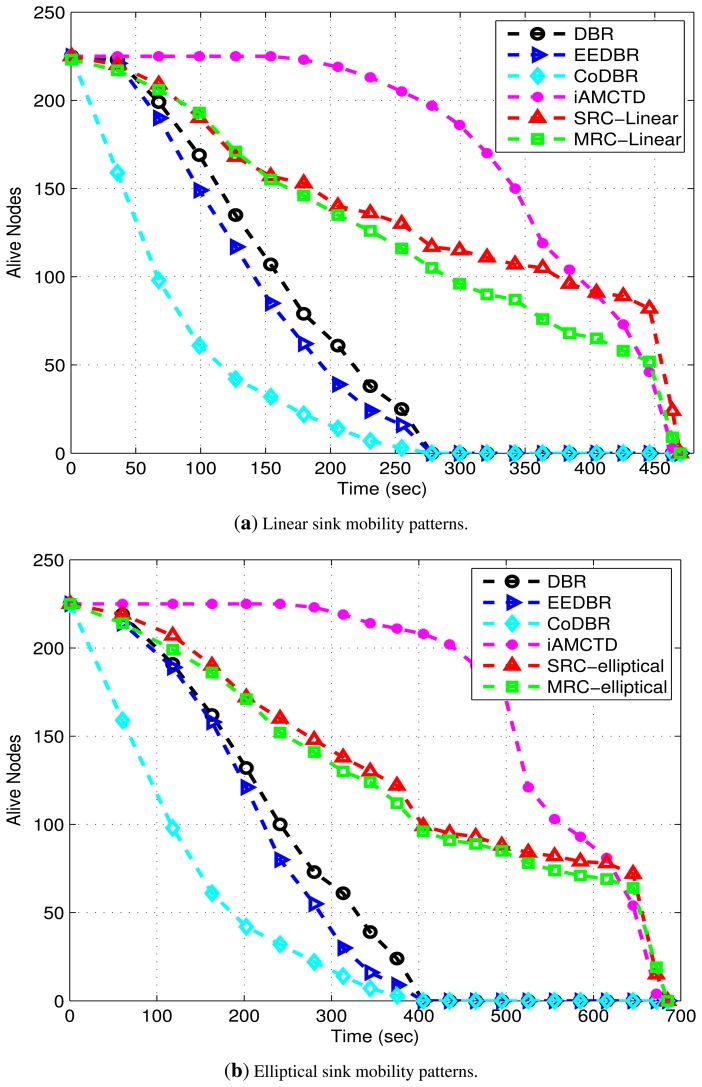
Lifetime with linear and elliptical sink mobility patterns.

As shown in Figure 10, in DBR, EEDBR and CoDBR, node density decreases sharply with time. The instability period is better in DBR as compared to EEDBR as there is a gradual increase in energy consumption. When network becomes sparse, number of neighbors decreases quickly which causes network instability. In DBR and CoDBR, low-depth nodes die at an earlier stage due to huge data forwarding and constant *d_th_*. DBR and CoDBR neglects both residual energy of nodes as well as link state in terms of SNR, whereas, EEDBR neglects link state which badly affects network throughput of these protocols. Number of dead nodes sharply increases with time in EEDBR as there is high load on high energy nodes because of considering residual energy as a routing metric. In CoDBR, network density decreases at the fastest rate because of cooperative routing being performed. Cooperative routing involves high energy consumption because of multiple transmissions and receptions of same data packet. Stability period (time up till which all nodes in the network are alive) of iAMCTD is highest because of optimal *d_th_* selection. Also, in iAMCTD, only source node transmits data to its next hop neighbor which conserves energy and results in improved stability period. Although due to implementation of rule 4, *d_th_* selection is more optimized in DEADS-SRC and DEADS-MRC as it requires only one-hop neighbor information instead of complete network density information, however, data is transmitted from source node as well as relay node(s) to the next hop. So, it is consuming 2 (SRC) to 3 (MRC) times more transmission and reception energy than iAMCTD due to which stability period is compromised. This shows the tradeoff between network stability period and reliability.

**Figure 11:**
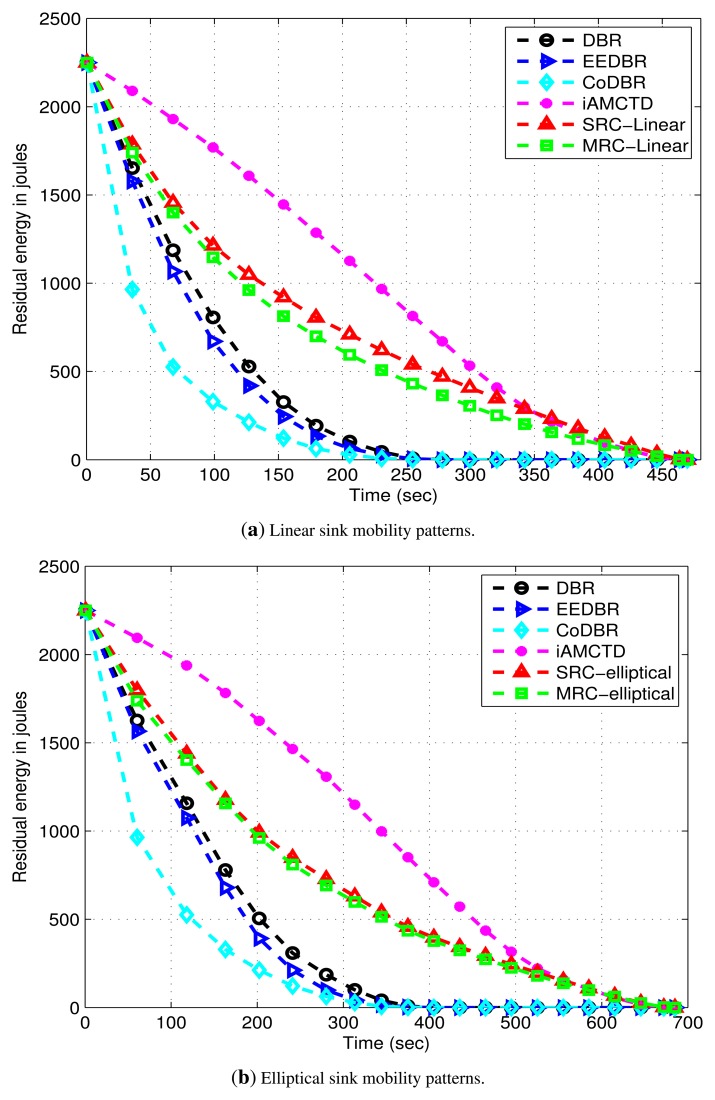
Residual energy possessed by the networks with linear and elliptical sink mobility patterns.

DBR shows better energy management than EEDBR as shown in Figure 11. In DBR, network residual energy steadily decreases as total number of eligible neighbors drops off with network density. Energy consumption of EEDBR is the highest among all other protocols due to frequent selection of high energy nodes. Moreover, higher energy consumption in DBR, EEDBR and CoDBR is because of reactive routing being performed in these protocols. Although CoDBR attains data reliability and high throughput, however, energy consumption is highest because of cooperative routing in the network as well as flooding resulting from fixed *d_th_*. iAMCTD attains a balanced residual energy throughout the network lifetime due to proper forwarder selection, optimal mobility patterns of courier nodes and maintenance of uniform network density. In DEADS-SRC and DEADS-MRC, high energy consumption is involved as compared to iAMCTD. This high energy consumption is because of cooperative routing. In the proposed protocol, as the network starts, nodes become active and start the sensing and transmission process. Initially all nodes are alive, each node finds appropriate relay and destination nodes and performs transmission and reception process due to which energy consumption is increased. After certain time, rate at which nodes become dead increases and energy consumption decreases. The reason behind this decreased energy consumption with increasing time is that nodes fail to find optimal relay nodes because of reduction in network density. As a result, chances of cooperative routing being performed by any source node are reduced which reduces energy consumption and most of the data is directly transmitted to the MSs or next hop node only. The proposed scheme achieves a much better energy management in spite of performing cooperative routing which involves high energy consumption.

**Figure 12:**
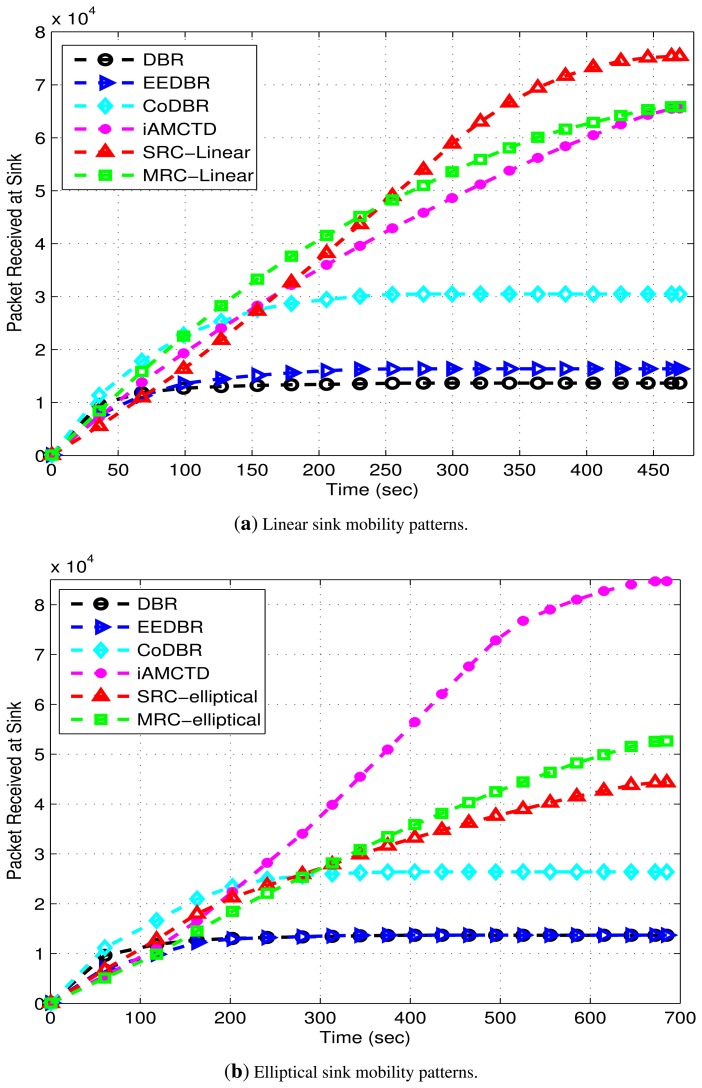
Packets received at MSs in the networks with linear and elliptical sink mobility‘patterns.

Figure 12 shows throughput of proposed and compared protocols. Throughput of DBR and EEDBR is almost constant after 100 s. This constant value is due to quick fall in network density. As network density decreases, nodes fail to find optimal forwarders for data forwarding due to which throughput attains a constant value. This behavior is unsuitable for both delay-tolerant and delay-sensitive applications. CoDBR attains high throughput due to cooperatively forwarding the data packets. Throughput of iAMCTD, DEADS-SRC and DEADS-MRC demonstrate the amount of threshold-optimized data obtained from reactive networks. In these protocols, unnecessary data transmission is avoided. In iAMCTD, throughput is high because of the selection of most optimal forwarder. This optimal forwarder is the one that possesses highest residual energy, least depth and depth difference (between source and next-hop node) and best link state at any particular time instant. Hence probability of data packet being corrupted by link impairments are reduced and high throughput is achieved. The proposed model for data routing attains optimal results with linear sink mobility as compared to elliptical sink mobility. Although, in DEADS-SRC and DEADS-MRC, only forwarder node's depth and residual energy are considered as selection parameters represented by Equations (11) and (15); however, improved results are achieved as compared to iAMCTD due to implementation of cooperative routing. If data received via direct route (source to *DS* node) is corrupted, it can be received successfully via an alternate route (source to *CC* to *DS* node). Along with this, implementation of MRC (diversity combining technique) at *DS* nodes further improves SNR of received data which results in high throughput. The highest throughput is achieved DEADS-SRC and DEADS-MRC with linear mobility pattern of MSs. Hence, this particular mathematical model performs the best with linear mobility pattern of MSs.

**Figure 13:**
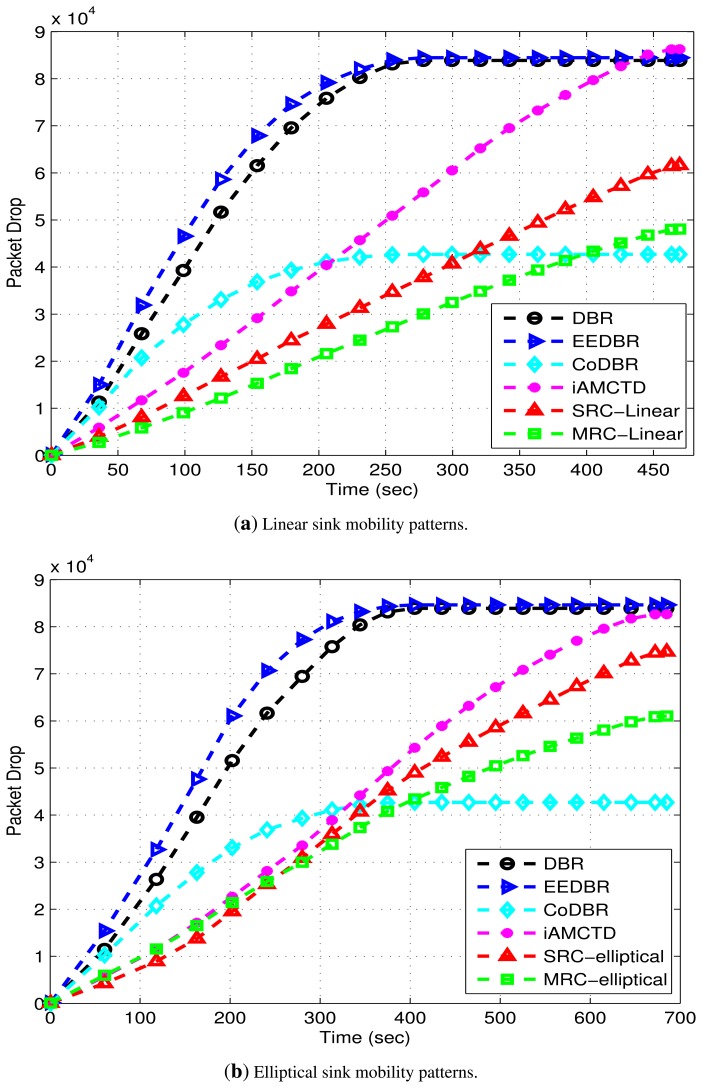
Packet drop attained in the networks with linear and elliptical sink mobility patterns.

Figure 13 show packet drop. High packet drop of DBR, EEDBR and iAMCTD is due to the availability of single link with poor link state most of the time. In CoDBR, packet drop is lesser because of cooperative routing. Although, iAMCTD considers link state of potential forwarder node before their selection, even then, the presence of redundant links as well as implementation of MRC (diversity combining technique) gives improved results and makes DEADS-SRC and DEADS-MRC more reliable. In CoDBR, DEADS-SRC and DEADS-MRC, packets are dropped only when no link (direct link to the *DS* node or via *CC* to *DS* node) is available for data forwarding or SNR of combined signal received at *DS* node (source to *DS* node, source to *CC* to *DS* node) is below an acceptable limit. In CoDBR, multiple transmissions of same data packet are performed before it is received by static sink. Effect of link impairments is high because of these multiple transmissions and as a result packet drop is high. Packet drop is higher in DEADS-SRC and DEADS-MRC with elliptical mobility pattern as compared to linear mobility pattern of MSs. This is because the proposed model is not optimized for elliptical mobility pattern. Whenever the condition(s) specified by *H_th_* and *S_th_* is met, packets are generated by nodes. Sink only accepts those packets that possess an acceptable SNR value and drops the remaining packets. In DEADS-SRC and DEADS-MRC with elliptical mobility pattern of MSs, data packets have to traverse multiple hops in order to get received at sink because of which their probability of being erroneous is high which results in packet drop. On the other hand, MSs are available for data reception mostly at one hop distance in linear sink mobility scenario because of which packet drop is reduced. Table 2 shows that packet drop rate of DEADS-SRC and DEADS-MRC is lowest with linear mobility pattern of MSs.

**Figure 14:**
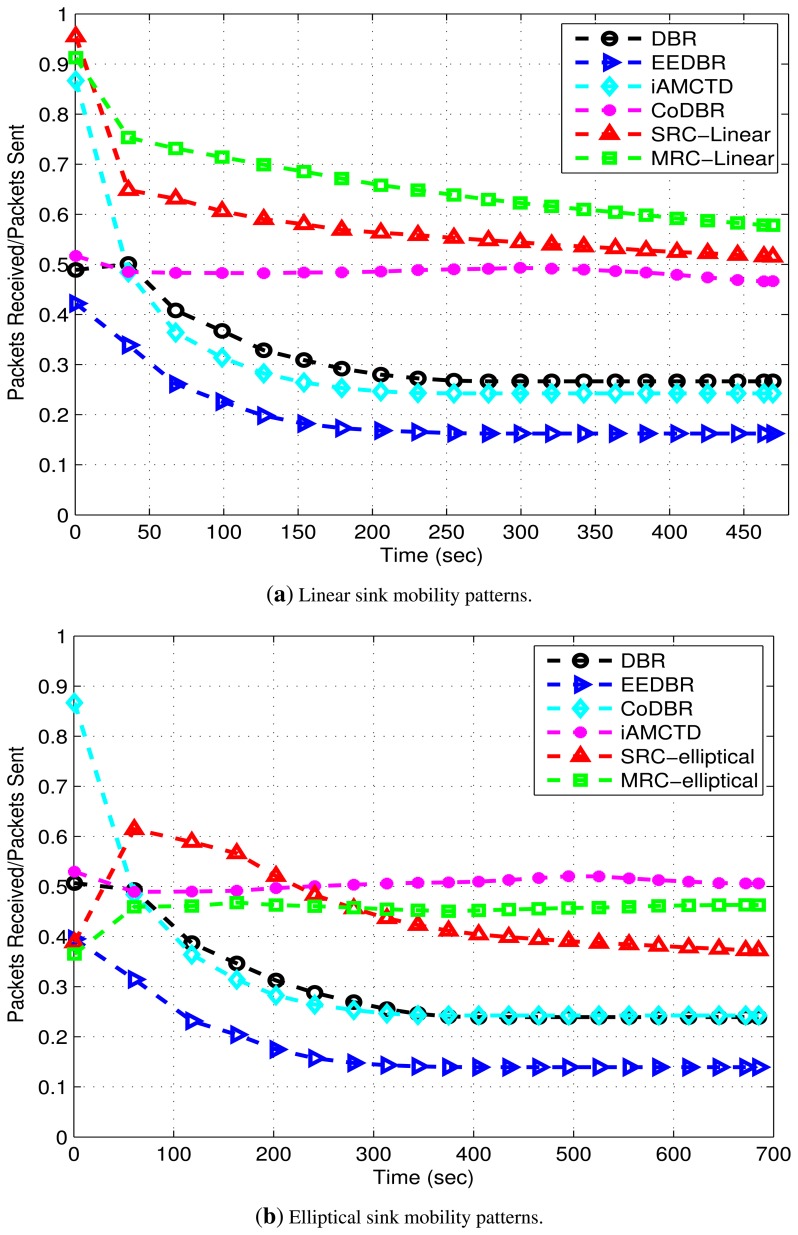
PAR attained in the networks with linear and elliptical sink mobility patterns.

PAR is shown in Figure 14. DBR and EEDBR have the lowest PAR as the number of packets received at sink are less due to single link having poor link state most of the time. iAMCTD shows higher PAR than DBR and EEDBR as it selects forwarder nodes on the basis of their respective link's SNR. This results in high throughput and increased PAR. Initially CoDBR attains higher PAR as compared to DBR, EEDBR and iAMCTD which later at decreases as throughput decreases. PAR is higher in DEADS-SRC and DEADS-MRC with linear mobility pattern of MSs as the number of packets received at sink in this scenario is high and packet drop is low.

Table 2 shows the improvement attained by our proposed protocol with respect to network lifetime, throughput and packet drop. Zero indicates that both protocols have same value for that specific evaluation metric. Negative value indicates reduced value of a particular metric with respect to compared protocol. Network lifetime of the proposed scheme is same as iAMCTD but outperforms DBR and EEDBR. Highest throughput and lowest packet drop is achieved by DEADS-SRC and DEADS-MRC with linear mobility pattern of MSs.

### 6.1. Performance Tradeoffs Made by DEADS

DEADS is a reactive routing protocol in which data is transmitted only when certain predefined thresholds are met. Hence it is not suitable for applications where continuous data monitoring and reception are required.

DEADS achieves improved network lifetime by introducing sink mobility in the network. In DEADS, cooperative routing is performed and high throughput is achieved at the cost of increased energy consumption and control overhead. Two to three times more energy consumption is involved when cooperative routing is performed in a network as shown in Figure 11. Along with this, control packet exchange for selection of cooperative partner nodes increases control overhead.

An optimized value of *d_th_* in accordance with Equation (4) is attained at the cost of control packet exchange. Nodes require their one-hop neighbor information in order to select their *d_th_*.

There is a tradeoff between the network's stability period and data reliability. The stability period is compromised in order to attain reliable data transfer as shown in Figure 10. Data reliability refers to the authenticity of received data and it is affected by the damage caused by link impairments. The greater the effect of link impairments on the received data, the less reliable it is and increased are the chances of it being dropped by receiving node and vice versa. Whereas, the link state varies from node to node. Higher energy consumption causes nodes to die at the start of the network compromising stability period, however, because of transmission via alternate paths data reliability is achieved.

Reliable data transmission results in high throughput, as shown in Figure 12 because chances of successful data reception at sink are increased. Hence, there is a tradeoff between throughput and energy consumption.

Reliable data transfer is attained at the cost of increased processing overhead (in terms of MRC and AF) and increased delay. Data is transmitted from source to relay and destination node. Relay node after performing AF at the received data forwards it to destination node. Destination node performs MRC at the received copies, selects appropriate relay and destination node and then forwards the data. In all this processing, a certain overhead is involved. Delay is also introduced because of which the proposed protocol is not suitable for delay sensitive applications.

DEADS achieves reduced packet drop as shown in Figure 13 at the cost of multiple transmissions. Greater number of transmissions increase reliability at the cost of extra energy consumption. Tradeoffs are shown in Table 3.

## 7. Conclusions

In this paper, we proposed a *DS* based cooperative routing algorithm along with sink mobility for UWSNs. The proposed algorithm operates in three phases: the neighbor selection phase, *DS* and *CC* set formation phase, and threshold based data sensing and routing phase. During the neighbor selection phase, nodes select *d_th_*, find neighbors, and attain their depth and residual energy information. During the *DS* and *CC* set formation phase, source nodes utilize information attained in previous phase and select their respective *DS* and *CC* nodes for cooperative routing. In the threshold based data sensing and routing phase, source nodes sense data on the basis of pre-defined threshold value and forward it to their cooperative partner nodes. We evaluated the performance of our proposed protocol by comparing it with DBR, EEDBR, and iAMCTD in terms of network lifetime, throughput, PAR and energy consumption. Results show better performance of our proposed work as compared to the existing works in terms of the selected performance metrics.

## Figures and Tables

**Table 1:** Analysis of existing depth-based routing protocols.

**Protocol**	**Depth Threshold** (**d***_th_*)	**Sink Mobility**	**Routing**	**Parameter for Forwarder Node Selection**
**DBR**	Fixed	Static sinks (on water surface)	Multi-hop routing	Depth
**EEDBR**	Fixed	Static sinks (on water surface)	Multi-hop routing	Depth and residual energy
**CoDBR**	Fixed	Static sinks (on water surface)	Cooperative routing	Depth
**iAMCTD**	Variable (on basis of network density information)	Mobile courier nodes	Multi-hop routing	Depth, residual energy and link's SNR
**DEADS (proposed)**	Variable (on basis of one-hop neighbor information)	Mobile sinks	Cooperative routing	Depth and residual energy

**Table 2:** Performance comparison of DEADS-SRC and DEADS-MRC with counterpart techniques.

**Evaluation Metric**	**Protocol**	**DEADS-SRC**		**DEADS-MRC**	

		**Linear**	**Elliptical**	**Linear**	**Elliptical**
**Network lifetime**	DBR	40%	45%	40%	45%
	EEDBR	40%	45%	40%	45%
	CoDBR	40%	45%	40%	45%
	iAMCTD	0	0	0	0

**Throughput**	DBR	76%	69%	75%	71%
	EEDBR	76%	69%	75%	71%
	CoDBR	63%	51%	60%	55%
	iAMCTD	6.4%	−23%	−1%	−13%

**Packet drop**	DBR	−41%	−29%	−51%	−31%
	EEDBR	−37.5%	−25%	−48%	−27%
	CoDBR	−15%	1.5%	−30%	−1.5%
	iAMCTD	−35%	−22%	−46%	−24%

**Table 3:** Performance tradeoffs made by routing protocols

**DEADS**	**Modification over Existing Protocols**	**Advances Achieved**	**Price to Pay**
**DEADS-SRC**	Single relay cooperative routing along with sink mobility	High throughput (Figure 12), reduced packet drop (Figure 13), optimized *d_th_* (rule 4)	High energy consumption (Figure 11), stability period compromised (Figure 10), control and processing overhead

**DEADS-MRC**	Multiple relay cooperative routing along with sink mobility	High throughput (Figure 12), reduced packet drop (Figure 13), optimized *d_th_* (rule 4)	High energy consumption (Figure 11), stability period compromised (Figure 10), control and processing overhead
